# Supplementary material for: In situ characterization of nanoparticle biomolecular interactions in complex biological media by flow cytometry
Source: Nat Commun. 2016 Nov 15;7:13475. doi: 10.1038/ncomms13475 (PMC5116075; doi:10.1038/ncomms13475)
Supplement: Supplementary Information — Supplementary Figures 1-27, Supplementary Tables 1-3 and Supplementary References [file ncomms13475-s1.pdf]

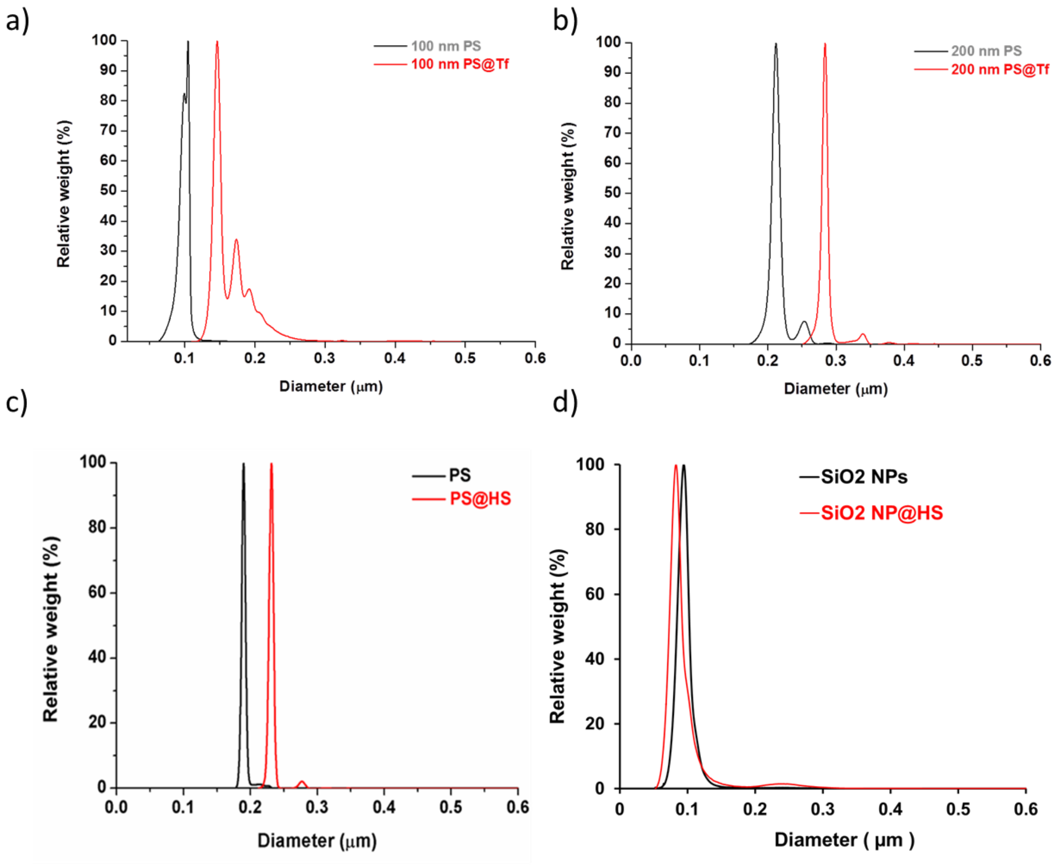

Supplementary Figure 1. Nanoparticle characterization. Size distribution of bare and Tf coated PS NPs (100 nm (a) and 200 nm (b)). After adsorption of the Tf corona a shift toward higher apparent diameter is observed in DCS as a consequence of the change in sedimentation properties of PS@Tf, compared to the pristine PS NPs. Size distribution of bare and 80 % HS corona coated PS NPs (200 nm) (c) and SiO_2 NPs (100 nm) (d). After formation of the HS corona a shift toward higher or lower apparent diameter is observed in DCS for PS and SiO_2 NPs respectively, as a consequence of the change in sedimentation properties of the NP@HS compared to the pristine NPs.

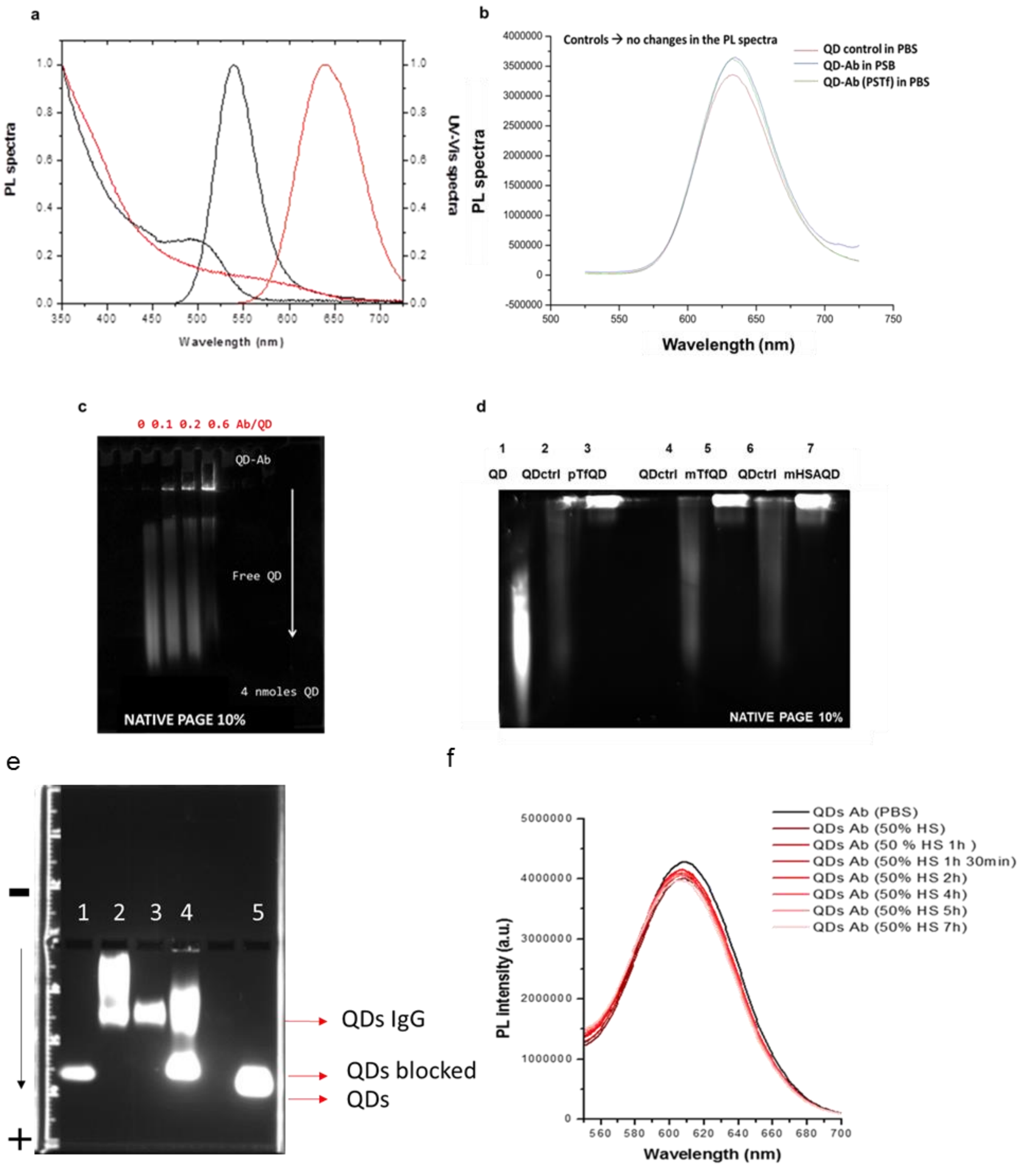

Supplementary Figure 2. Characterization of QDs. a) Green emitting MPA-CdTe QDs with maximum emission at 530 nm (35 % QY) and orange emitting MPA-CdTe QDs with maximum emission at 630 nm (36 % QY) were synthesized and used during all the experiments. b) Photoluminescence spectra of the QDs in PBS, QDs functionalized with antibodies (QDAb) in PBS buffer and dispersed in a solution of PS NPs in PBS buffer. The covalent immobilization of the antibody onto the QD is characterized by the QD electrophoretic mobility in a 10 % native PAGE gel. c) Different ratios of Ab conjugated per QD have been analysed. 0.6 Ab/QD is the ratio used for all the experiment. d) QDs free (1), QDs control (QDs activated with EDC and Sulfo-NHS and blocked with 4-Aminophenyl β-D-galactopyranoside, 2, 4 and 6) and QDs functionalized with Ab (3, 5 and 7) are run in 10 % NATIVE-PAGE. e) The covalent immobilization of the antibody onto the QD is characterized by the QD electrophoretic mobility in a 1 % agarose gel (50V, 30 min). QDs control (QDs activated with EDC and Sulfo-NHS and blocked with 4-Aminophenyl β-D-galactopyranoside) (1), QDsAb with

different ratios of Ab conjugated per QD have been analyzed (2-4). 0.6 Ab/QD is the ratio used for all the experiment (3) and QDs free (5). f) Fluorescence spectra of QDs in serum over time. Photoluminescence spectra of immuno-QDs dispersed in 50 % HS over time (1 h, 1.5 h, 2 h, 4 h, 5 h and 7 h). Photoluminescence spectra of immuno-QDs dispersed in HS were recorded over time and no substantial variation in the intensity of the photoluminescence emission peak was observed.

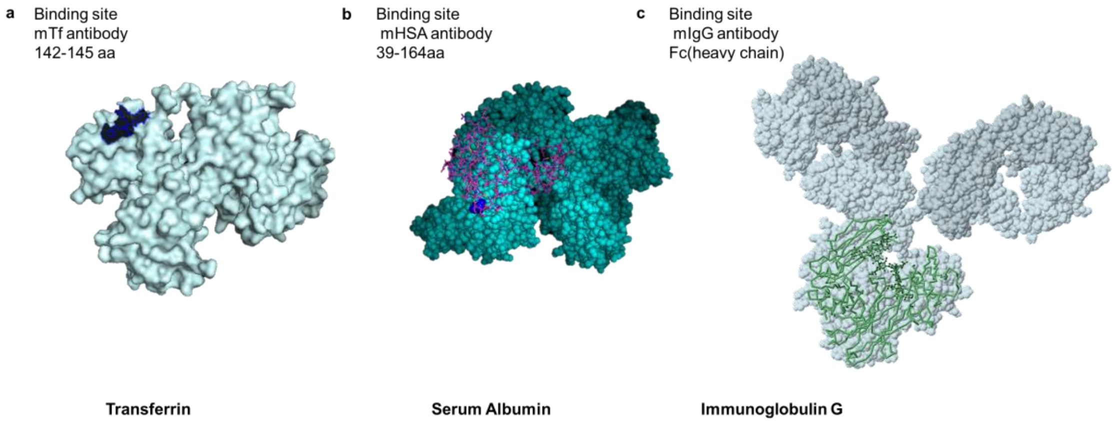

Supplementary Figure 3. Epitope recognized by the different antibodies used in this study. Protein structure and epitope recognized for: a) Tf (epitope in dark blue) b) HSA (epitope in purple) and c) IgG (epitope in green). Structures reprinted from PDB.

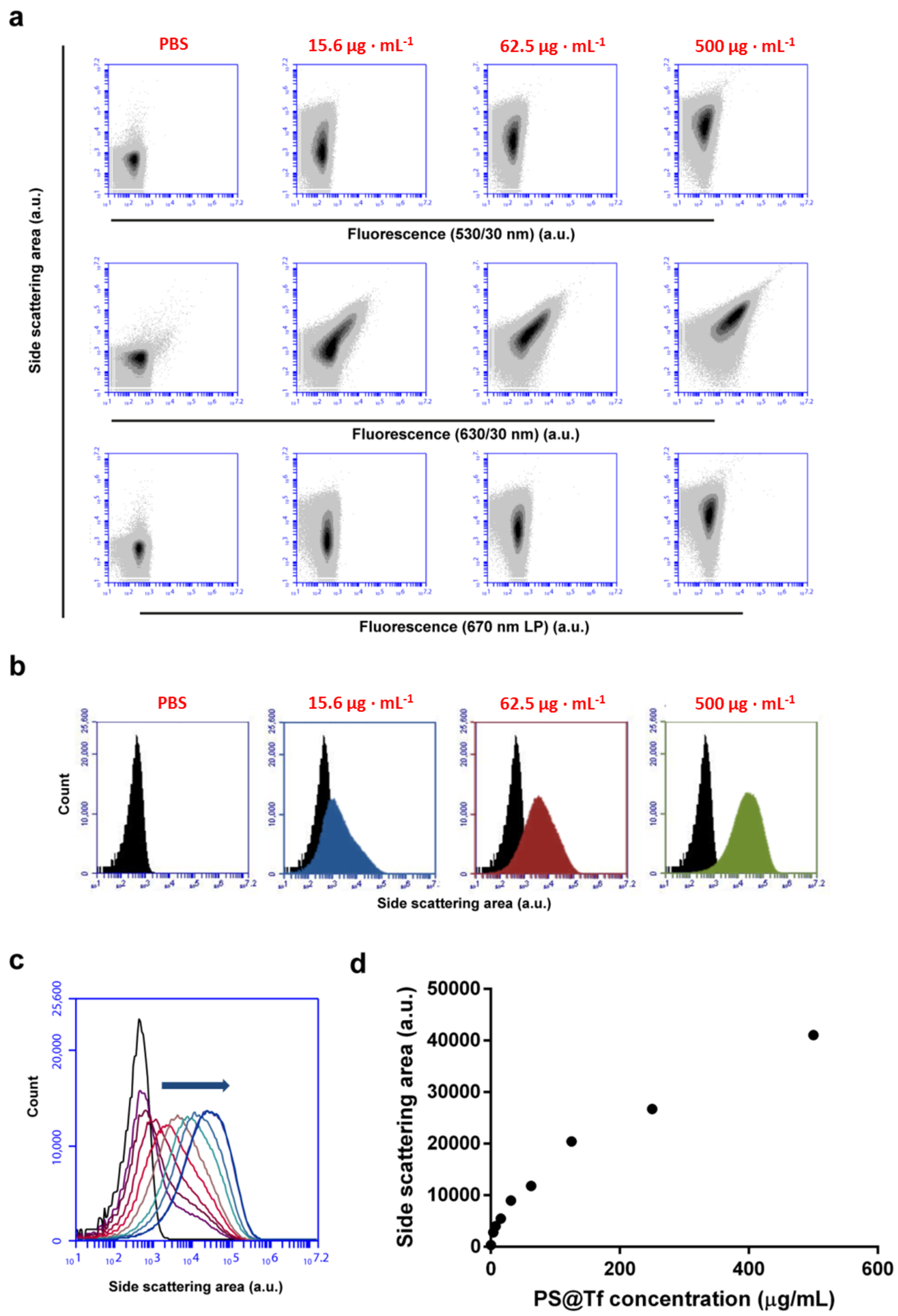

Supplementary Figure 4. NP concentration analysis on flow cytometry for 200 nm PS@Tf NPs. a) Scatter density plots of side scattering area signal versus fluorescence signal in three different channels (530/30 nm, 630/30 nm and 670 nm LP) for different concentrations of PS@Tf NPs. b) overlap of the side scattering distribution of PBS (background noise) and PS@Tf samples at three different concentrations. c) Overlap of the side scattering distribution of increasing concentration of PS@Tf NPs. d) Variation of side scattering signal in function of the 200 nm PS@Tf NPs. A linear trend can be observed for the lowest NP concentrations, while at high concentration values the curve deviates from the linearity. All epitope mapping experiments performed with 200 nm PS NPs were carried out using a NP concentration of 500 µg·mL^{-1}.

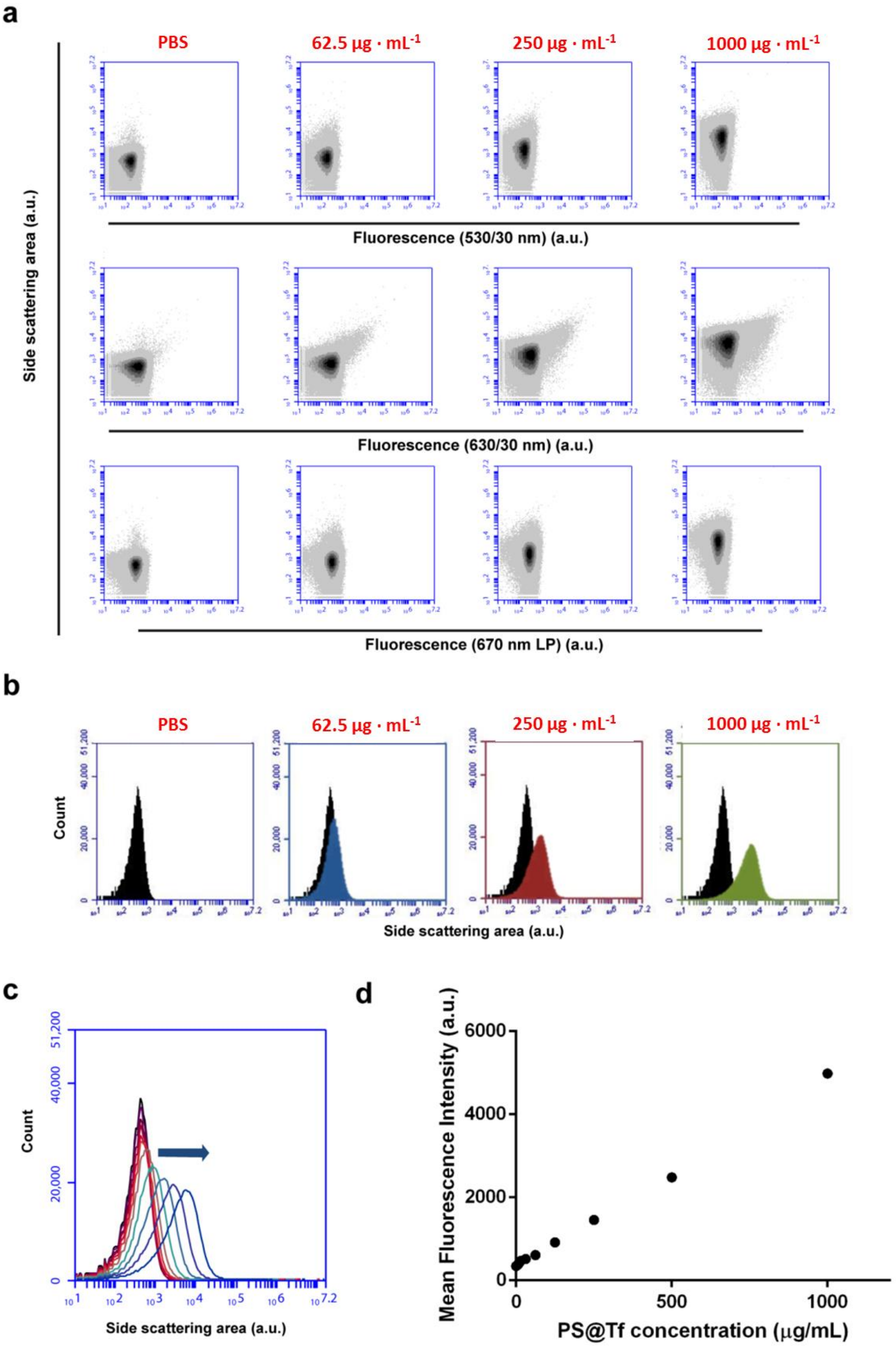

Supplementary Figure 5. NP concentration analysis on flow cytometry for 100 nm PS@Tf NPs. a) Scatter density plots of side scattering area signal versus fluorescence signal in three different channels (530/30 nm, 630/30 nm and 670 nm LP) for different concentrations of PS@Tf NPs. b) Overlap of the side scattering distribution of PBS (background noise) and PS@Tf samples at three different concentrations. c) Overlap of the side scattering distribution of increasing concentration of PS@Tf NPs. d) variation of side scattering signal in function of the 100 nm PS@Tf NPs. A linear trend can be observed in the range of concentrations used. All epitope mapping experiments performed with 100 nm PS NPs were carried out using a NP concentration of 1000 µg·mL^{-1}.

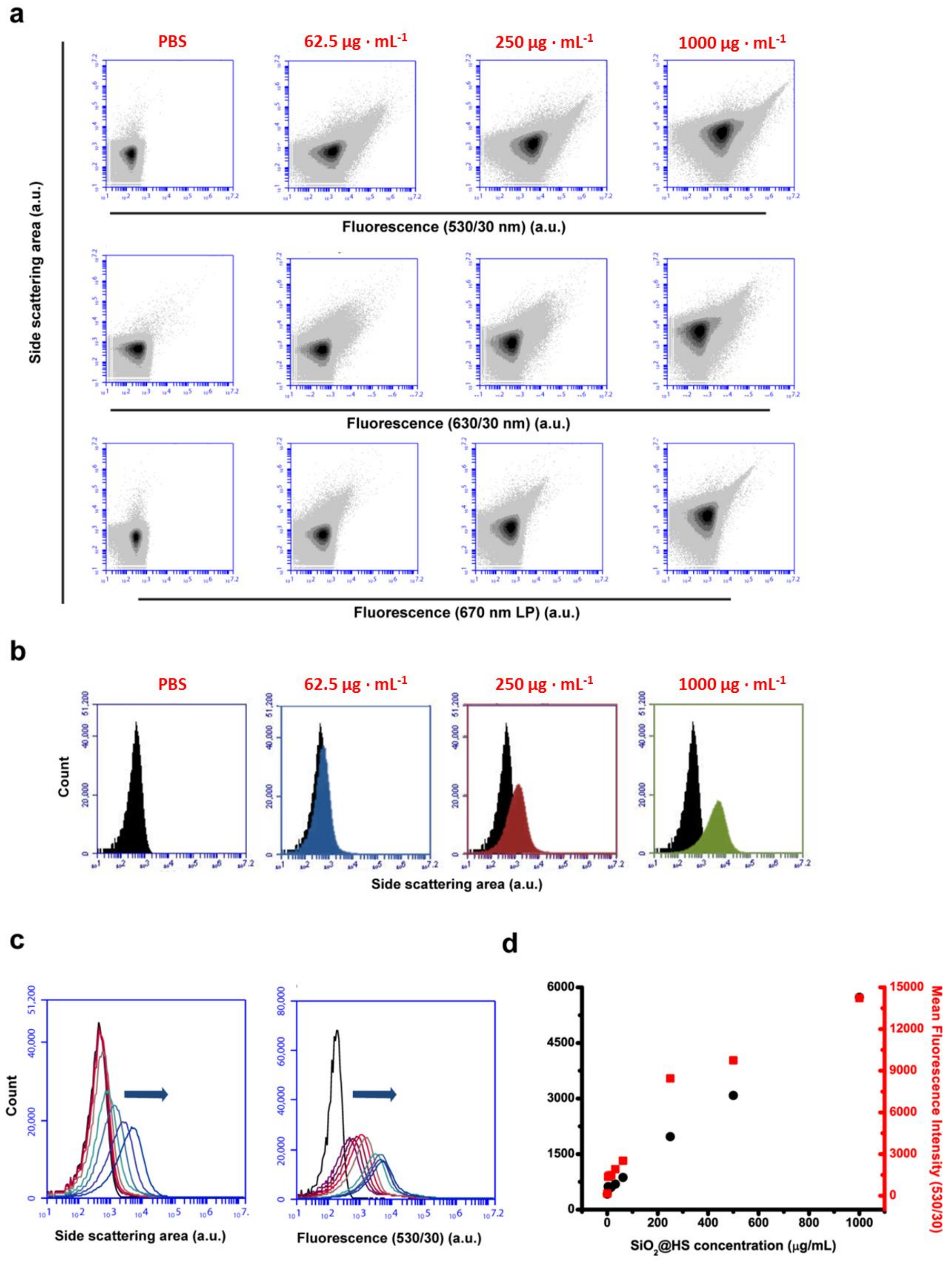

Supplementary Figure 6. NP concentration analysis on flow cytometry for fluorescence 100 nm SiO_2@HS NPs. a) Scatter density plots of side scattering area signal versus fluorescence signal in three different channels (530/30 nm, 630/30 nm and 670 nm LP) for different concentrations of SiO_2@HS NPs. b) Overlap of the side scattering distribution of PBS (background noise) and SiO_2@HS samples at three different concentrations. c) Overlap of the side scattering distribution of increasing concentration of SiO_2@HS NP (left), and of the fluorescence signal in the green channel (530/30 nm) of increasing concentrations of SiO_2@HS NPs. The increase in side scattering resembles the one observed for the same size of PS NPs (100 nm), confirming that the capability of

distinguishing the NPs from the background noise in the swarm regime is mainly size dependent. In the fluorescence channel NPs can be distinguished from background even at the lowest concentration values utilized, indicating that with fluorescent NPs it is possible to lower the number of NPs in the swarm. d) Variation of side scattering and fluorescence (530/30 nm) signal in function of the 100 nm SiO_2@HS NPs. A linear trend can be observed in the range of concentrations used. For consistency with the rest of the work presented in this paper, all the epitope mapping experiments were performed with a NP concentration of 1000 µg·mL^{-1}. At this concentration the FITC fluorescence of the NP (mainly visible in the 530/30 nm channel) is spilling over considerably in the 630/30 nm channel. For this reason the data analysis of the experiments of SiO_2@HS and PS@HS NPs was performed considering the 670 LP channel, where no signal compensation is needed.

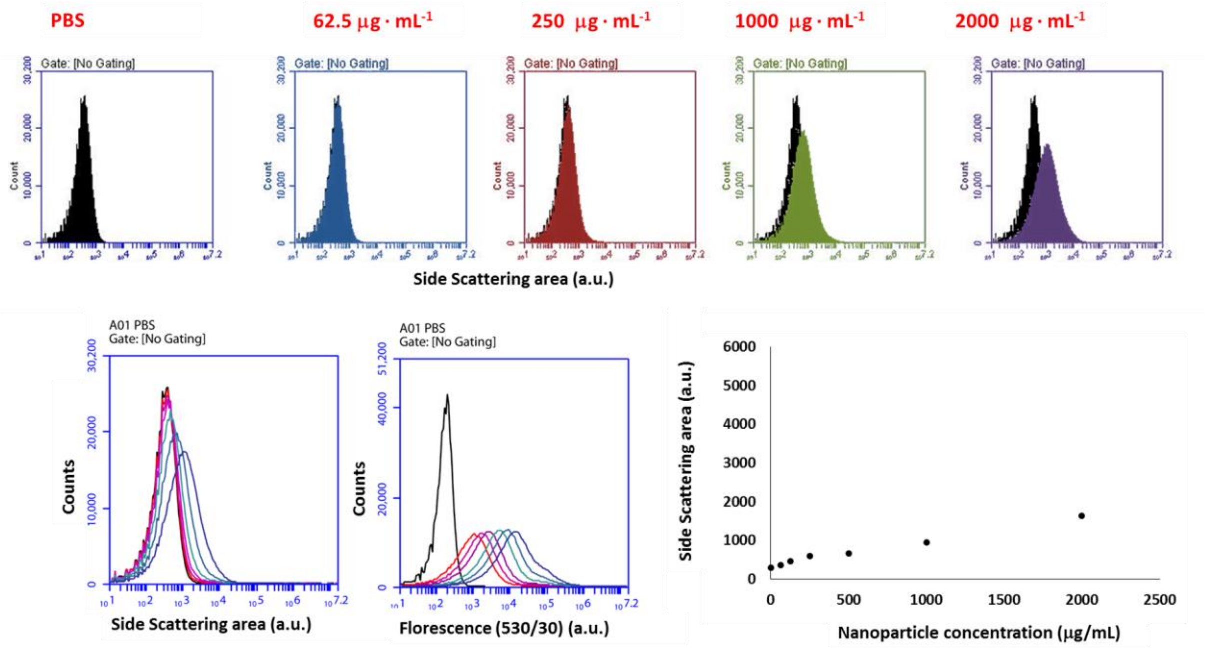

Supplementary Figure 7. NP concentration analysis on flow cytometry for fluorescence 50 nm SiO_2@HS NPs (FTIC). a) Overlap of the side scattering distribution of PBS (background noise) and SiO_2@HS samples at different concentrations. b) Overlap of the side scattering distribution of increasing concentration of SiO_2@HS NP (left), and of the fluorescence signal in the green channel (530/30 nm) of increasing concentrations of SiO_2@HS NPs. The increase in side scattering resembles the one observed for the same size of PS NPs (50 nm), confirming that the capability of distinguishing the NPs from the background noise in the swarm regime is mainly size dependent. In the fluorescence channel NPs can be distinguished from background even at the lowest concentration values utilized, indicating that with fluorescent NPs it is possible to lower the number of NPs in the swarm. c) Variation of side scattering signal in function of the 50 nm SiO_2@HS NPs concentration.

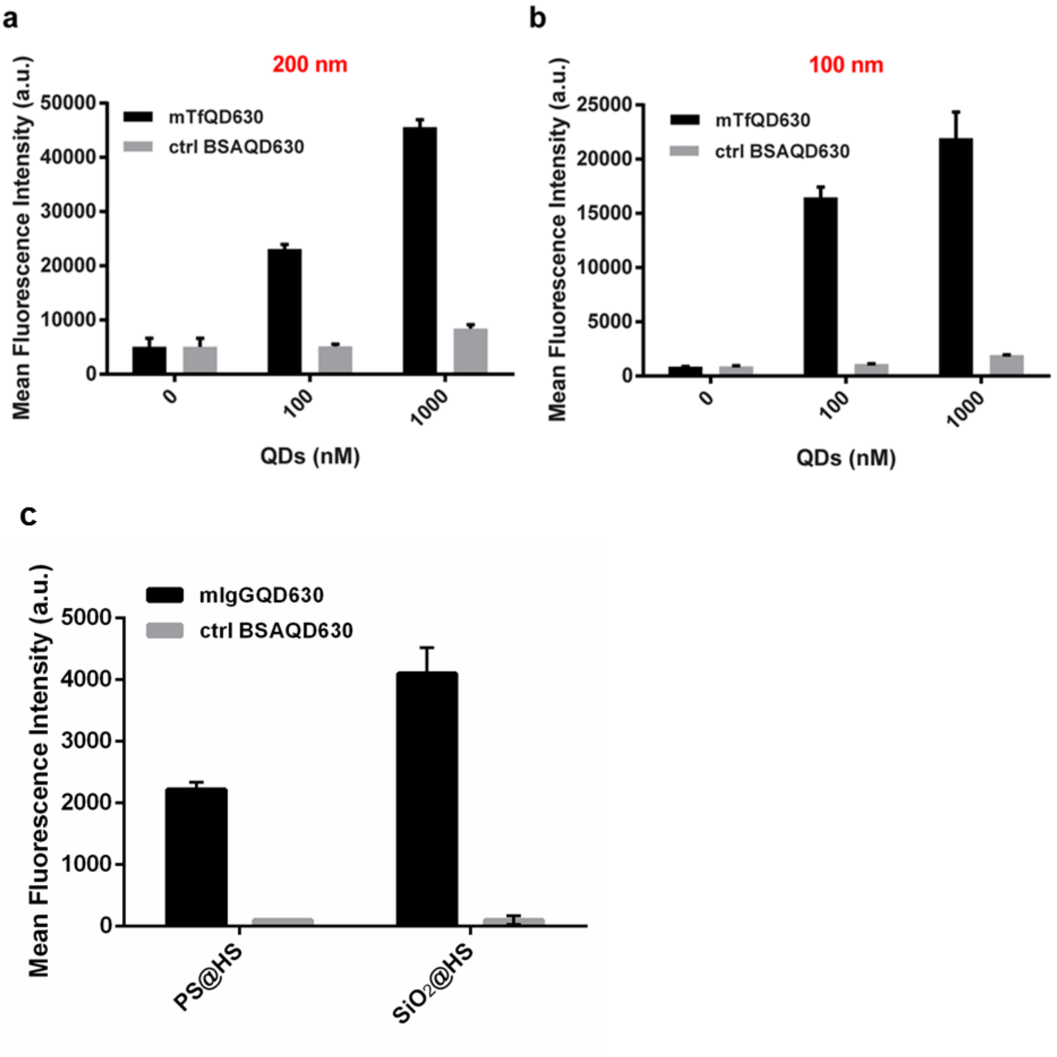

Supplementary Figure 8. Specificity of the biorecognition assay. The specificity of the immuno-QD binding has been determined by studying the interaction of the PS@Tf NPs with QDs functionalized with bovine serum albumin (BSA). The BSA functionalized QDs did not bind significantly to the PS@Tf, indicating that the contribution of unspecific binding of the mTfQDs to PS@Tf NPs in the determination of the amount of exposed epitopes is negligible. Fluorescence signal at 630/30 nm for 200 nm (a) and 100 nm (b) PS@Tf NPs incubated with anti-Tf conjugated QDs ($mTfQD_{630}$, black) and bovine serum albumin conjugated QDs, used as a control for non-specific binding (ctrl $BSAQD_{630}$, grey). c) Fluorescence signal at 670 LP for 200 nm PS@HS (left) and 100 nm SiO_2@HS (right) NPs incubated with anti-IgG conjugated QDs ($mIgGQD_{630}$, black) and bovine serum albumin conjugated QDs, used as a control for non-specific binding (ctrl $BSAQD_{630}$, grey). The BSA functionalized QDs did not bind significantly to the biomolecular corona of the NPs, indicating that the contribution of unspecific binding of the mIgGQDs in the determination of the amount of exposed epitopes is negligible.

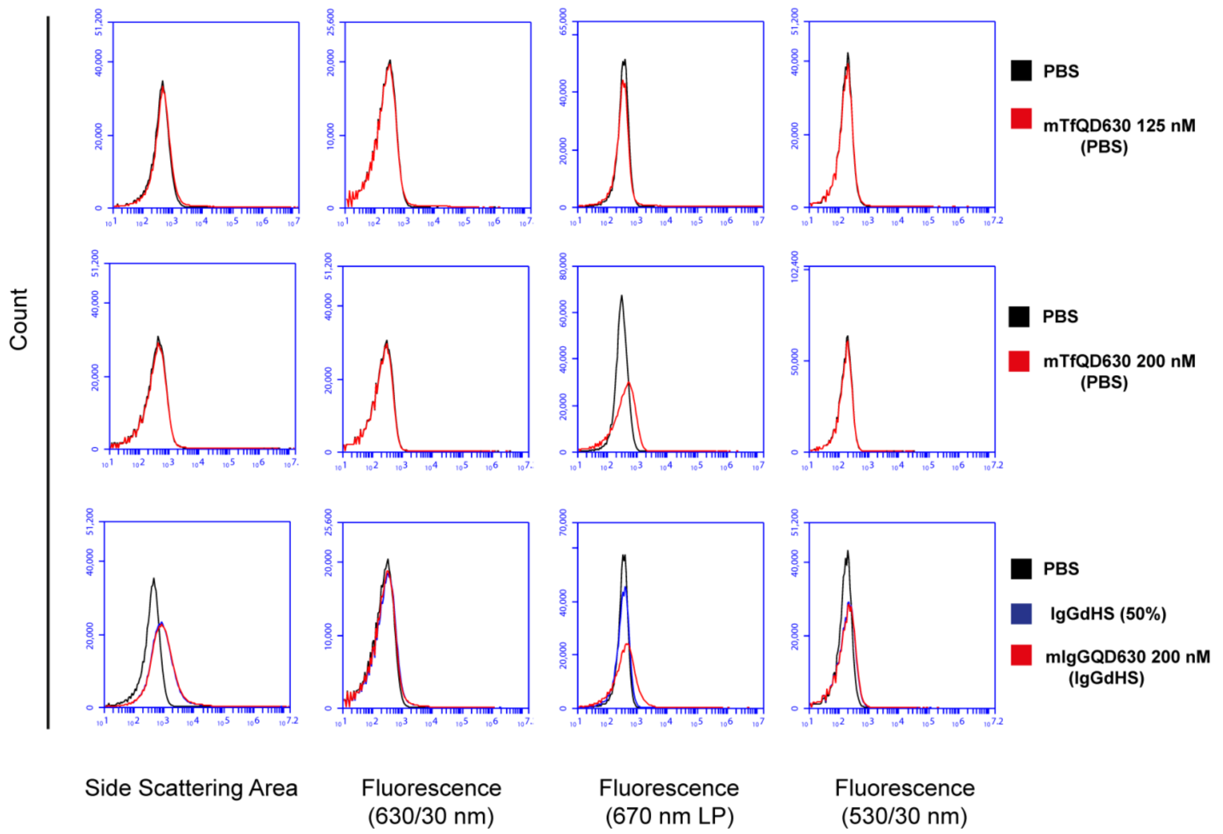

Supplementary Figure 9. Side scattering and fluorescence distributions of mTfQDs in PBS and IgGQDs in IgGdHS. In the range of concentration used for the epitope mapping experiment, the side scattering signal of the QDs does not overcome the background noise (PBS, black and IgGdHS blue). A shift is detected in the fluorescent channel 670 nm LP, but it remains negligible compared to the shift of the labeled NPs. The low signal of the QDs can be explained in terms of size. At the size of the QDs (ca. 4 nm), a higher number of QD NPs would be required in the swarm to be visible. This allowed us to “see” just the QDs bound to the PS and SiO_2 NPs, but not the QDs free in solution, explaining the similarity between the experiments performed washing the samples, and the *in situ* ones (Figure 3 in the manuscript).

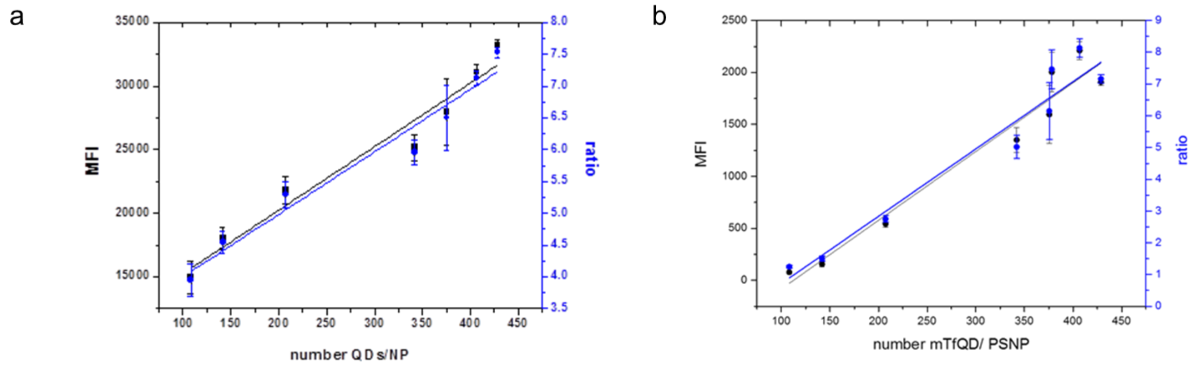

Supplementary Figure 10. Calibration of the flow cytometry output. Fitting analysis of mean fluorescence intensity MFI (ratio of MFI of QD labeled NPs and MFI of NPs in blue) vs number of mTfQD attached to the PS@Tf NPs surface. a) The fluorescence intensity increases linearly with the number of QDs labeling the proteins on the PS NPs surface. Linear fitting analysis for MFI y = ax + b, a = 49.87, b = 10273 and R-square 0.952. Linear fitting analysis for MFI ratio y = ax + b, a = 0.001, b = 3.02 and R-square 0.952. In order to get semi-quantitative information from the flow cytometry analysis, the flow cytometry signal was calibrated using fluorescence spectroscopy. First, a set of samples of known QD concentration was measured in the spectrofluorimeter to get a calibration curve for the QD signal in fluorescence spectroscopy (Figure S7). Then, PS@Tf NPs titrated with $mTfQD_{630}$ were washed to remove the unbound immunolabels and measured in parallel with both flow cytometry (Figure 1 in the main text) and fluorescence spectroscopy (figure S9). The photoluminescence peak of the immunolabeled samples was compared with the QD calibration curve, under the assumption that the total fluorescence of the sample with a certain number of QDs is equivalent to the amount of fluorescence intensity of a solution of known QDs concentration. This allowed us to estimate the number of immuno-QDs on the NP surface. The number of immunolabels per NP for each sample can then be correlated with the corresponding flow cytometry signal. This calibration enables the estimation of the number of exposed epitopes for all the samples titrated with the same QD batch. An analogue calibration procedure was performed for the green emitting QDs (QD_{530}, Figure S20). b). Calibration of 200 nm PS@Tf NPs by green emitted QDs functionalized with monoclonal antibody anti-Tf ($mTfQD_{530}$). Fitting analysis of mean fluorescence intensity MFI (ratio of MFI of QD labeled NPs and MFI of NPs in blue) vs number of mTfQD attached to the PS@Tf NPs surface. Linear fitting analysis for MFI y = ax + b, a = -743.5, b = 6.610 and R-square 0.947. Linear fitting analysis for MFI ratio y = ax + b, a = -1.4, b = 0.0212 and R-square 0.937.

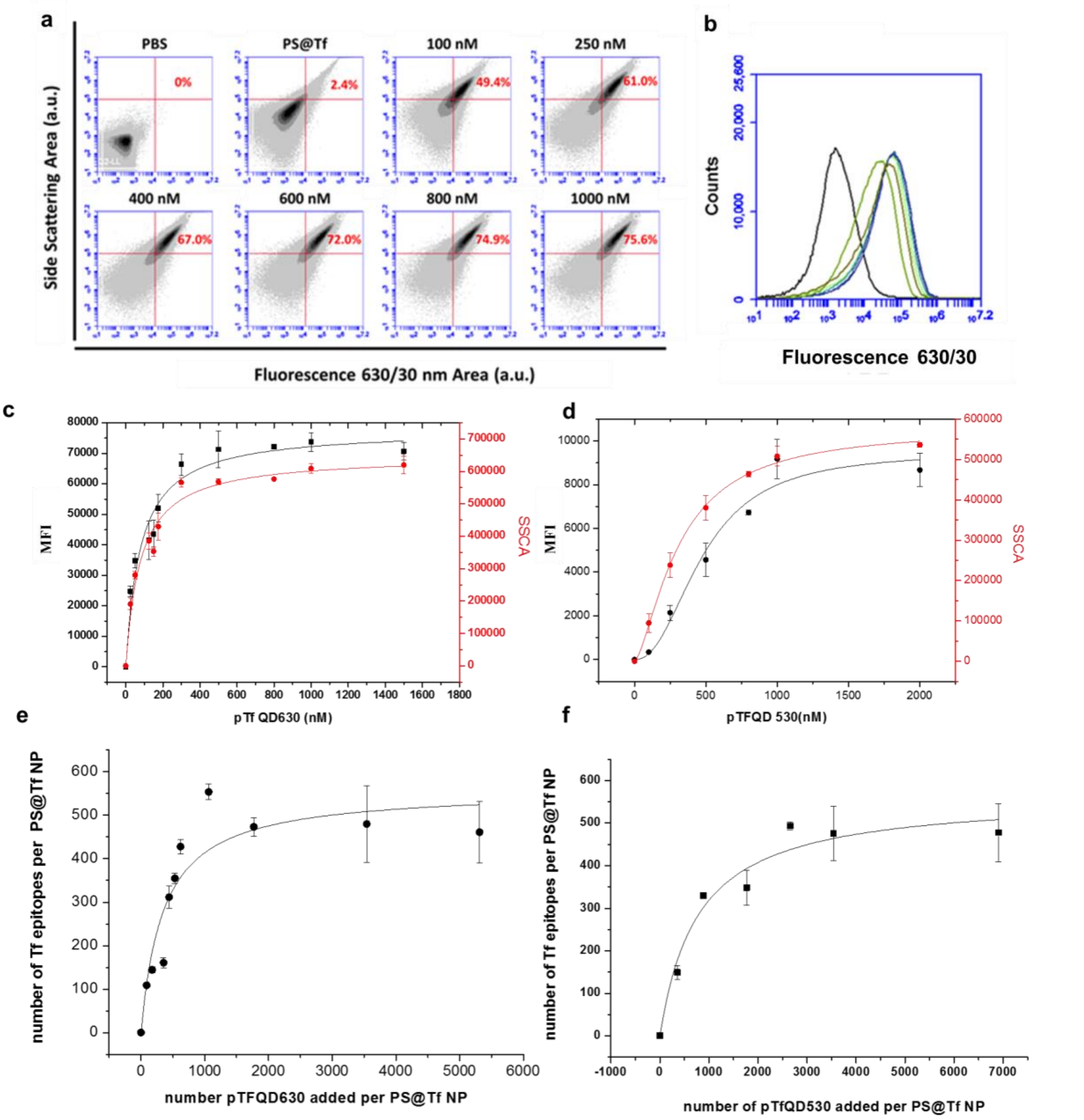

Supplementary Figure 11. Epitope mapping of PS@Tf NP by polyclonal antibody. Binding analysis of the pTfQD onto 200 nm PS@Tf NPs (with orange emitting QDs, left graphs c and e, and green emitting QDs, right graphs d and f) by flow cytometry and epitope quantification by fluorescence spectroscopy. The 200 nm PS@Tf NPs model has also been studied using QD conjugated to a polyclonal antibody against human Tf. The results follow the same trend observed in the case of the monoclonal antibody, i.e. an increase in fluorescence and side scattering proportional to the amount of QDs interacting with the protein epitopes on the surface. The variation in side scattering and fluorescence is higher than the one observed with the monoclonal, indicating a higher number of recognized Tf epitopes (558 ± 57 epitopes for $pTfQD_{630}$).

a)

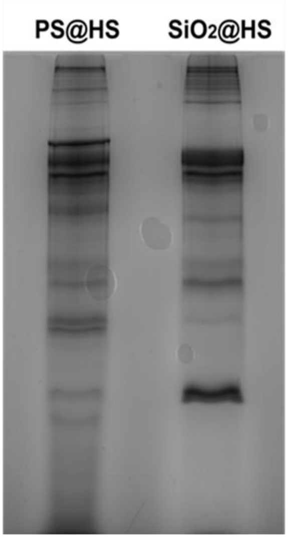

b)

Number of Tf molecules / NP		
	Theoretical	Micro-BCA assay
200 nm PS@Tf	2844	1530 ± 141
100 nm PS@Tf	711	459 ± 51

c)

Protein concentration		
	Mass protein/mass of NP	Number proteins/NP
200 nm PS@HS	0.119 ± 0.01	2340 ± 146
100 nm SiO_2@HS	0.076 ± 0.01	1470 ± 116

Supplementary Figure 12. Concentration of proteins adsorbed on 100 nm PS NPs, 200 nm PS NPs and 100 nm SiO_2 NPs. a) SDS-PAGE of 80 % HS corona coated PS NPs (200 nm) and SiO_2 NPs (100 nm). The difference in the bands suggests a different protein corona composition. b) The table shows a comparison between the theoretical calculation and the concentration determined experimentally by micro-BCA assay. c) Protein concentration in PS@HS and SiO_2@HS NPs samples determined by micro-BCA assay. The number of proteins per NP has been estimated by considering the molecular mass of HSA (66 kDa) as an average mass of the proteins that form the biomolecular corona.

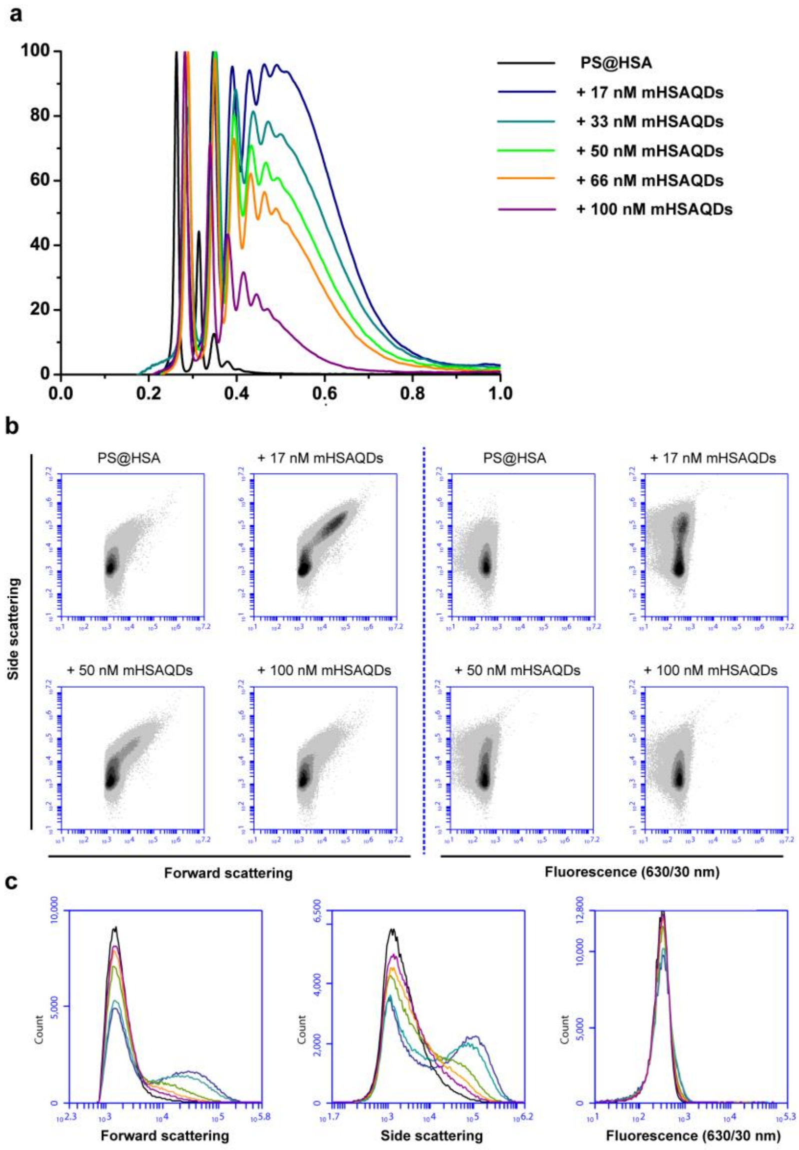

Supplementary Figure 13. Flow cytometry analysis of polydisperse samples. The unique characteristics of flow cytometry of monitoring at the same time scattering and fluorescence of the samples offers the possibility of using this technique to monitor at the same time fluorescence and state of dispersion of NPs. Example of analysis of a polydisperse sample (PS@HSA titrated with $mHSAQD_{630}$) in flow cytometry. a) DCS analysis of PS@HSA NPs titrated with $mHSAQD_{630}$. Multiple peaks corresponding to aggregates of NPs are detected. b) Scatter density plots of side scattering versus forward scattering (left) and versus fluorescence (630/30 nm, right) for some points of the titration curve. c) Forward scattering, side scattering and fluorescence distribution of PS@HSA NPs titrated with $mHSAQD_{630}$. The presence of aggregates of NPs, confirmed by differential centrifugal sedimentation (Figure a) is detected by flow cytometry in both the forward and side scattering channel (Figure b, c). Even in the swarm regime, flow cytometry analysis can be used to monitor at the same time the fluorescence and dispersion properties of NP samples.

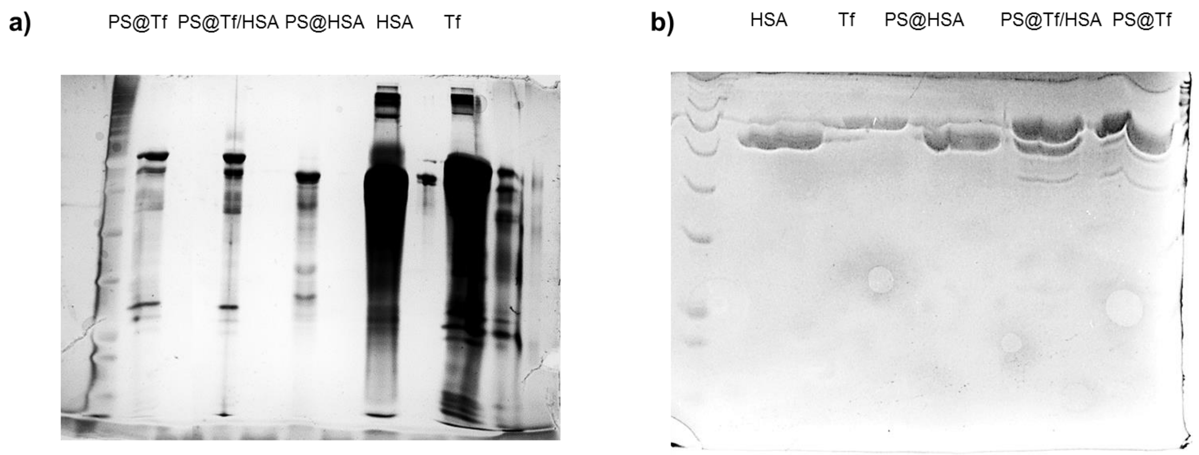

Supplementary Figure 14. Protein corona characterization by SDS polyacrylamide electrophoresis. SDS PAGE of PS@Tf NPs, PS@Tf/HSA NPs and PS@HSA NPs after silver staining (a) and Coomassie staining (b).

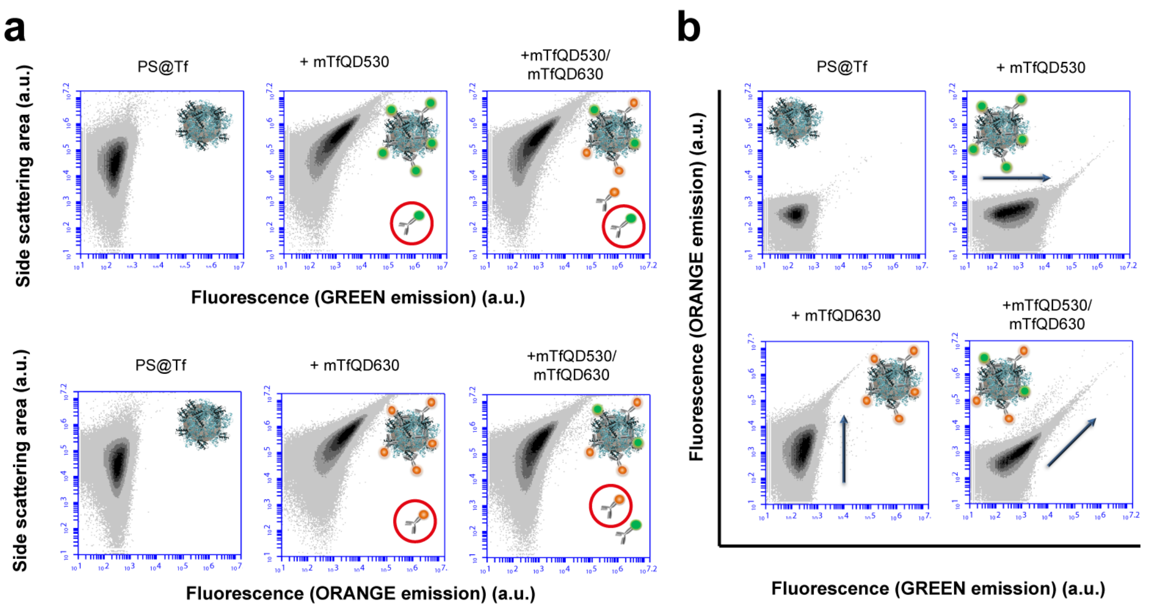

Supplementary Figure 15. Two colors flow cytometry epitope mapping of 200 nm PS@Tf NPs. To validate the multiplexing epitope mapping approach, we titrated 200 nm PS@Tf NPs with either orange emitting immuno-QDs (mTfQD$_{630}$), green emitting immuno-QDs (mTfQD$_{530}$) or a one to one mixture of the two a) Scatter density plots of side scattering versus fluorescence in the green channel (530/30 nm, top) and in the red channel (670 nm LP, bottom) for the PS@Tf NPs control (first column), for the PS@Tf NPs immunolabeled with either green emitting monoclonal antibody anti-Tf QDs (mTfQD$_{530}$) or orange-emitting monoclonal antibody anti-Tf QDs (mTfQD$_{630}$) (second column), and for PS@Tf NPs titrated with a mixture of both mTfQD$_{530}$ and mTfQD$_{630}$ (third column). b) Corresponding scatter density plots of fluorescence in the green channel versus fluorescence in the red channel. A fluorescence increase in the red or green channel is observed for the PS@Tf titrated with a mixture of both mTfQD$_{530}$ and mTfQD$_{630}$. As expected, when the NPs were titrated with a mixture of the two color immunolabels, we observed an increase in both the green (530/30 nm) and the red (670

nm LP) fluorescence channels. The number of epitopes (370 ± 10) detected summing the contribution of the green and the orange emitting immuno-QDs in the mixed sample matches the total amount of epitopes (364 ± 27) detected in a single color experiment (Figure 3 in the main text).

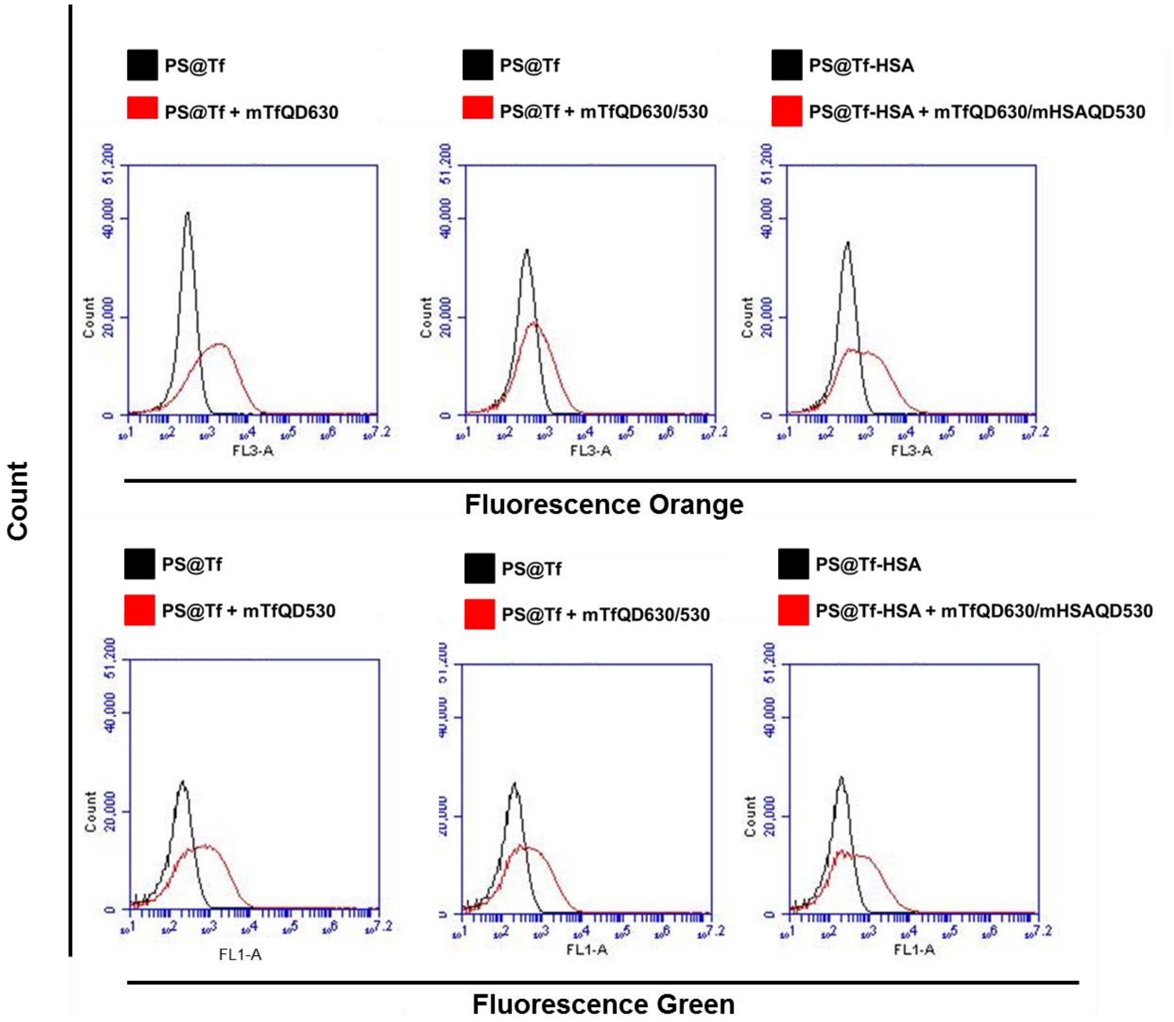

Supplementary Figure 16. Fluorescence distributions of PS@Tf and PS@Tf/HSA NPs control and immunolabeled. Upon immunolabeling it is possible to observe a shift towards higher fluorescence values, together with an increase in the width of the peak, which suggests the existence of a distribution of epitopes per NP in the sample, with some NPs having much higher amount of exposed epitopes than others.

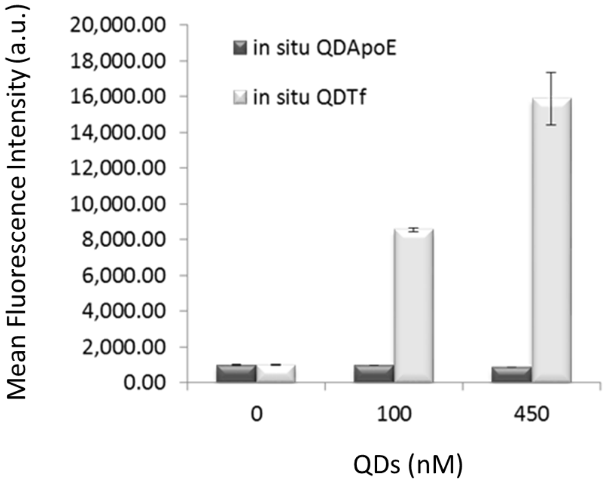

Supplementary Figure 17. Specificity of the biorecognition assay *in situ*. The specificity of the immuno-QD binding has been determined by studying the interaction of the PS@Tf NPs with QDs functionalized with a monoclonal antibody anti-Apolipoprotein E. The control functionalized QDs did not bind significantly to the PS@Tf, indicating that the contribution of unspecific binding of the mTfQDs to PS@Tf NPs in the determination of the amount of exposed epitopes is negligible. Fluorescence signal at 630/30 nm for 200 nm PS@Tf NPs incubated with anti-Tf conjugated QDs (light grey) and anti-ApoE conjugated QDs, used as a control for non-specific binding (dark grey). The experiment was carried out in situ, without removing the excess of QDs from the solution.

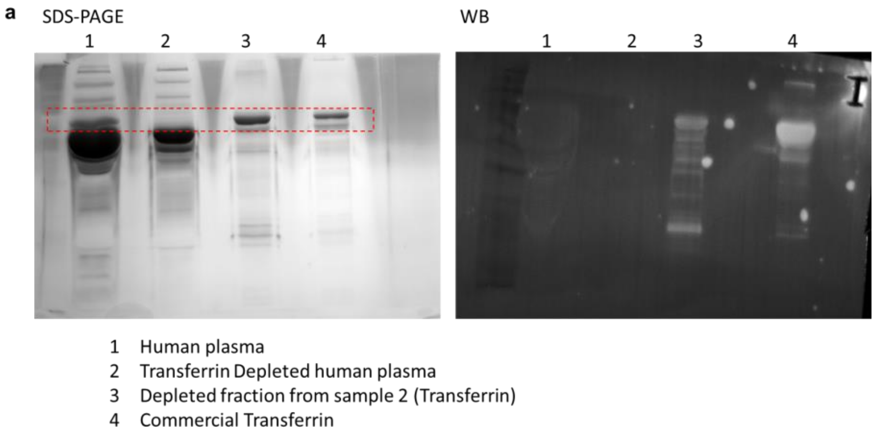

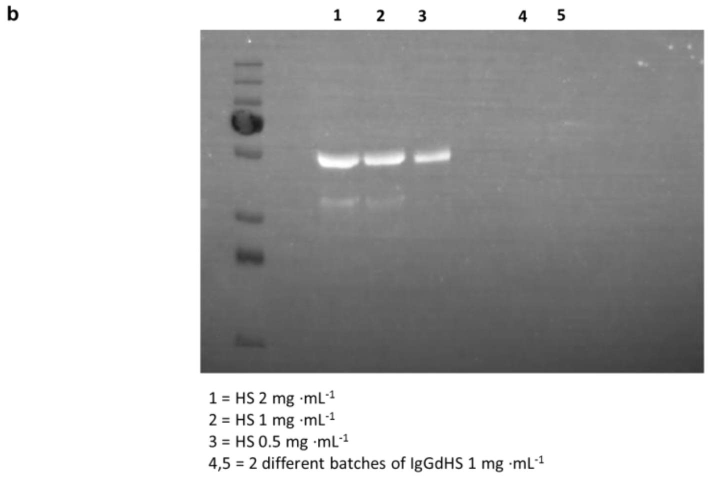

Supplementary Figure 18. Preparation and characterization of Tf depleted human plasma. Human plasma depleted of endogenous Tf was prepared by affinity chromatography using HiTrap NHS-Activated HP columns (GE Healthcare) activated with polyclonal antibody anti human Tf (Acris antibodies – R1112P), following the protocol described in Salvati et al[1]. The Tf depleted human plasma has been characterized by SDS-PAGE and Western Blot. For Western blot immuno-detection of residual Tf in the depleted serum, the HS before and after depletion (together with the proteins recovered in the corresponding eluted fractions as controls) were separated by SDS-PAGE (10 % polyacrylamide gels) (a). Then the separated proteins were transferred from the gel to PVDF membranes using a Mini-PROTEAN Tetra Trans-Blot Module under a constant voltage of 100 V for 1 hour. The membranes were then incubated at room temperature for 1 hour in “blocking solution” of 5 % skimmed milk in TBS-Tween (150 mM NaCl, 10 mM Tris HCl, 0.1 % Tween, pH 7.5). Afterwards, blots were incubated (4 °C, overnight) with 0.4 $\mu g \cdot mL^{-1}$ rabbit polyclonal antibody against human Tf in blocking solution (5 % skimmed milk in TBS-Tween) and subsequently washed three times for 15 minutes in TBS-Tween. After washing, the membranes were incubated with 0.1 $\mu g \cdot mL^{-1}$ of the anti-rabbit antibody in blocking solution for 1 hour at room temperature and washed three times for 15 minutes in TBS-Tween and once for 5 minutes in Millipore water. Specific antibody binding was visualized using a G:Box Chemi XT4 (Syngene) (b) Commercial IgG depleted human serum was characterized by SDS-PAGE and Wersten blot following the same protocol described above.

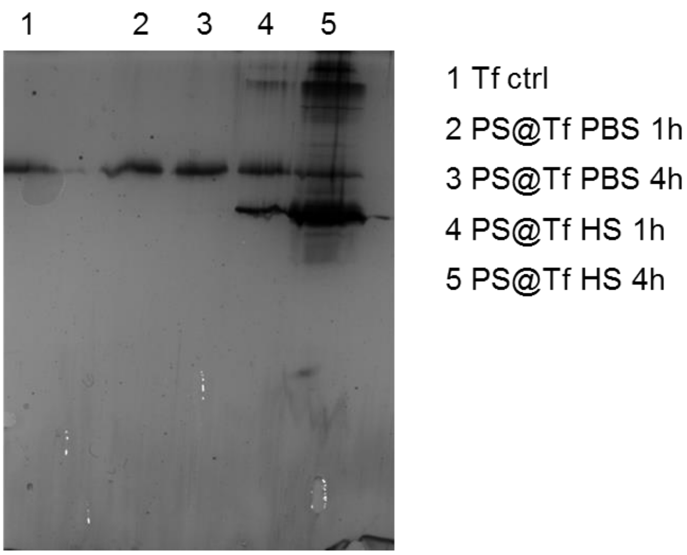

Supplementary Figure. 19. Protein corona characterization by SDS polyacrylamide electrophoresis. SDS PAGE of PS@Tf after 1 hour and 4 hours re-suspension in PBS (lines 2 and 3, respectively) and human serum (lines 4 and 5, respectively). Line 1 was loaded with Transferrin as a control.

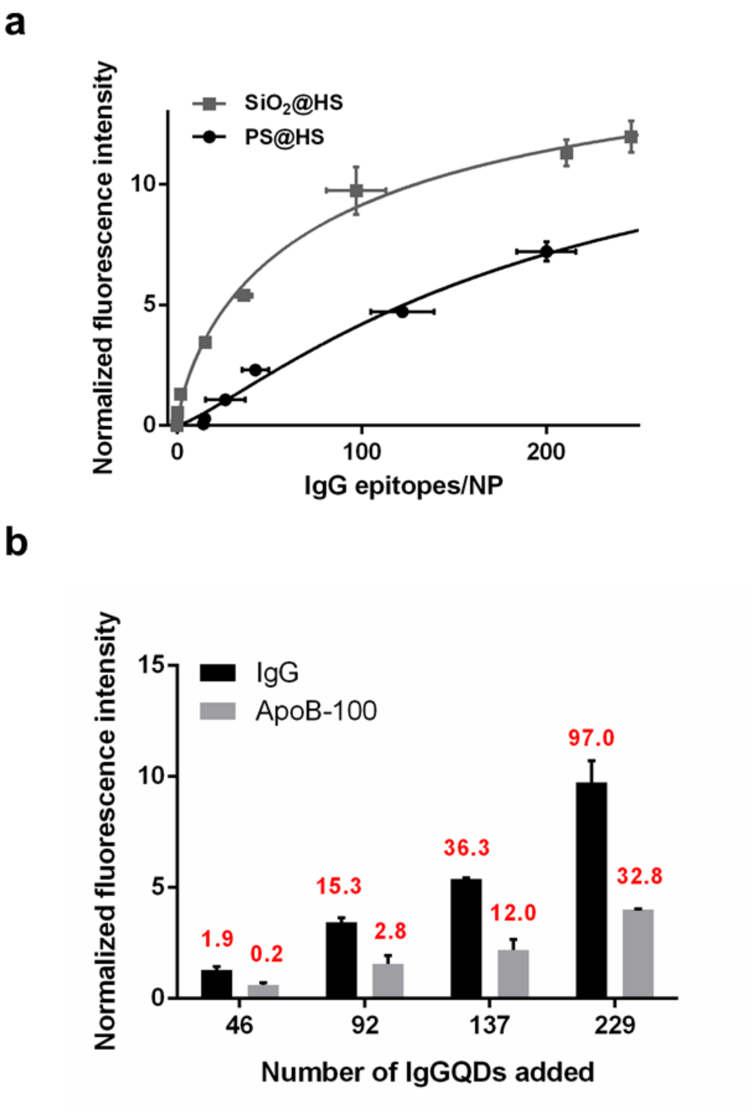

Supplementary Figure 20. Epitope mapping of hard corona. a) Correlation between the flow cytometry normalized fluorescence signal (ratio of MFI of QD labeled NPs and MFI of NPs) and the number of exposed epitopes for both PS@HS and SiO_2@HS NPs. The curve can be used to estimate the number of the exposed epitopes *in situ* in complex biological media (Figure 4 in the main text). b) Flow cytometry results of IgG (black) and ApoB-100 (grey) epitope mapping of SiO_2@HS NPs in PBS buffer. In order to provide another example of relevant epitope that can be mapped with the flow cytometry methodology, the 100 nm SiO_2@HS NPs were titrated with mApoB-100QD_{630}. For each concentration of immunolabels used, the number of exposed ApoB-100 epitopes is lower than the IgG epitopes. The amount of exposed epitopes for each point of the titration is indicated in red above the corresponding sample.

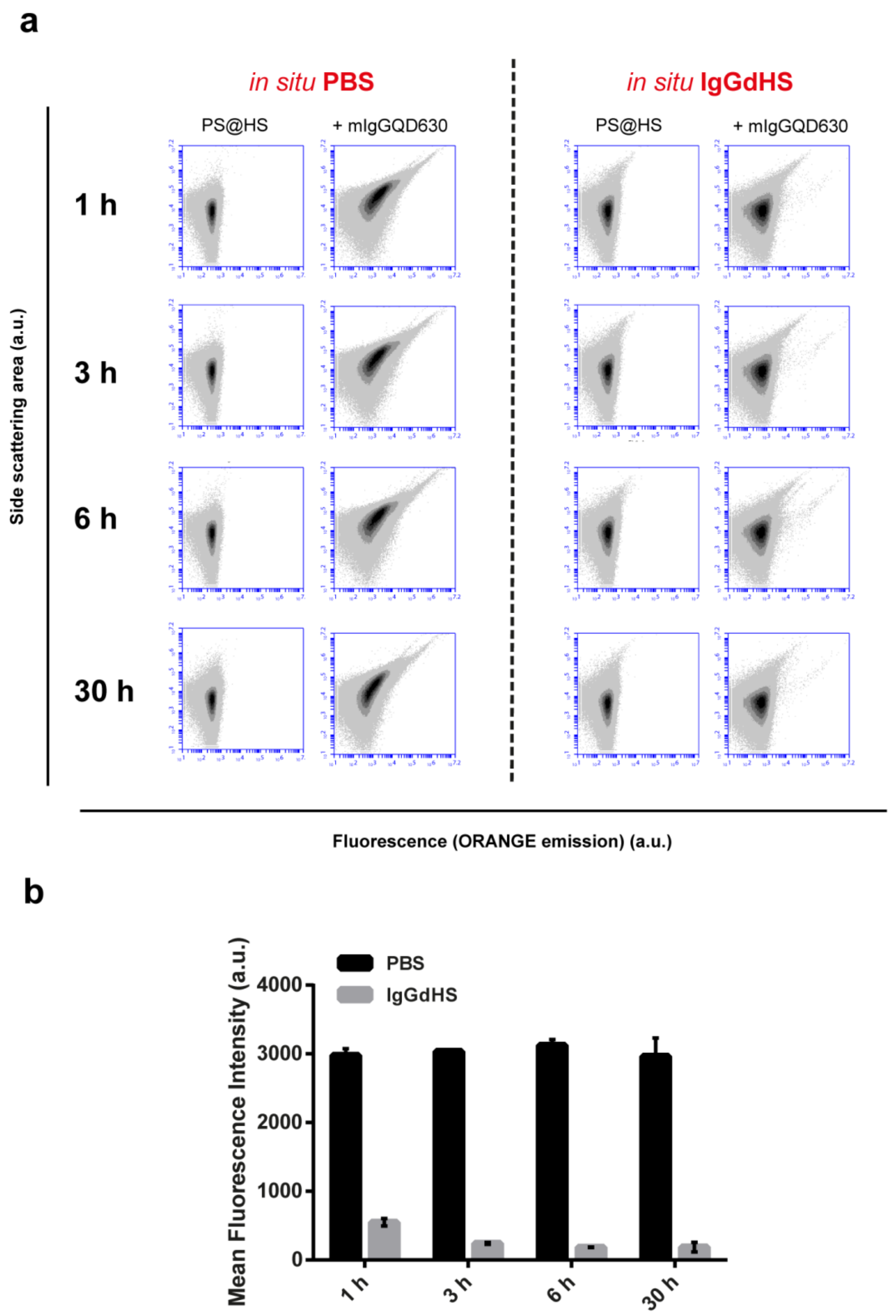

Supplementary Figure 21. IgG epitope mapping *in situ* in IgGdHS over time. *In situ* epitope mapping of PS@HS with IgGmQD$_{630}$ in PBS and in IgGdHS as a function of time. No increase in the fluorescence signal in IgGdHS was observed over 30 h time.

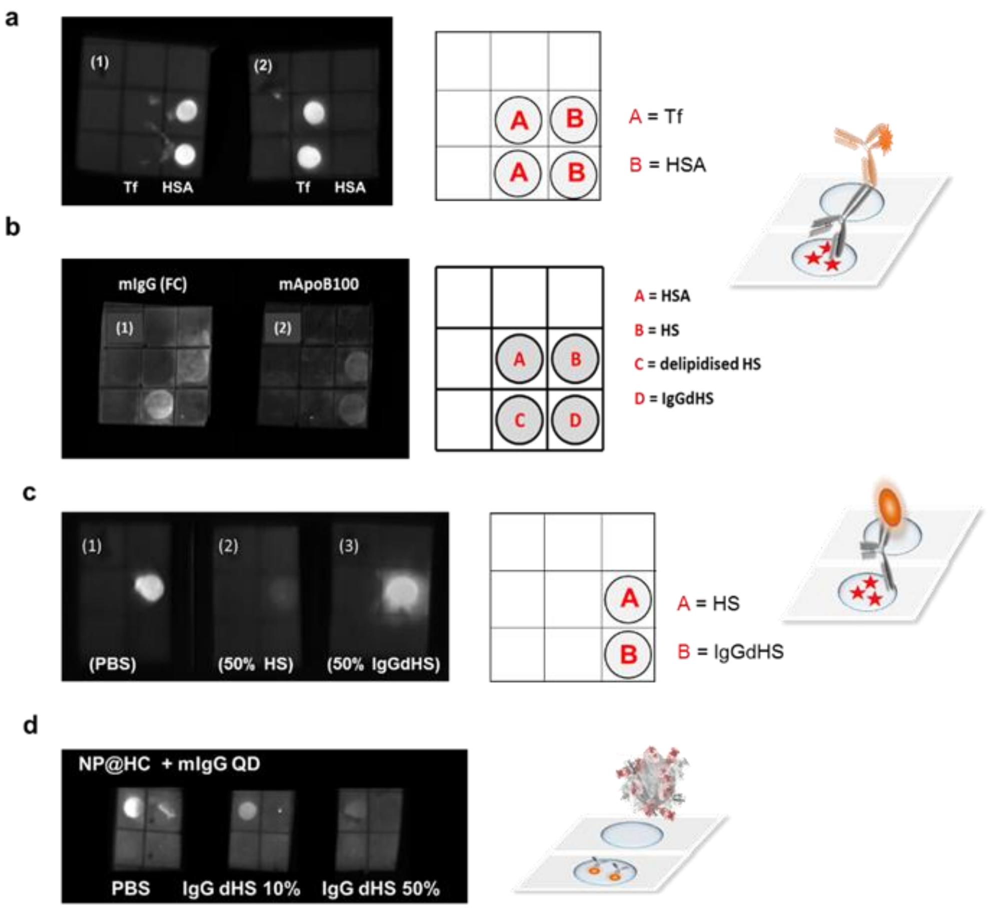

Supplementary Figure 22. Immuno dot blot assay. The antibody binding capacity and specificity were assessed by dot blot assay. The antigen of interest and a negative control were spotted on each of the polyvinylidene fluoride membranes (PVDF). The blots were blocked in 5 % skimmed milk in PBS for 1 hour at room temperature, washed three times in PBS and incubated with 1 μg·mL^{-1} of the corresponding antibody (monoclonal anti-Tf, monoclonal anti-HSA, monoclonal anti-IgG (Fc), and monoclonal anti-ApoB-100). After 1 h incubation with the antibody solution in PBS at room temperature, the blots were washed three times for 10 minutes, and incubated with a 0.5 μg·mL^{-1} solution of Alexa 488-labeled secondary antibody for 1 hour at room temperature. The blots were washed 5 times, dried and scanned for fluorescence using a G:Box Chemi XT4 (Syngene) to detect the presence of the primary antibody bound to the spots on the membranes. a) Immuno dot blot for monoclonal antibody anti-HSA (1) and monoclonal anti-Tf (2). Tf and HSA were spotted on each of the PVDF membranes at two different concentrations, 1 mg·mL^{-1} (top) and 0.2 mg·mL^{-1} (bottom). b) Immuno dot blots of monoclonal antibody against IgG (Fc) (1) and against ApoB-100 (2). The membranes were spotted with HSA as a negative control (45 mg·mL^{-1}, A), HS (77 mg·mL^{-1}, B), delipidised HS (58 mg·mL^{-1}, C) and IgG depleted HS (55 mg·mL^{-1}, D). c) The IgG (Fc) recognition by immuno-QDs in situ was also studied by dot blot analysis. Immuno dot blot analysis of $mIgGQD_{630}$. Human serum have been spotted on the PVC membrane. The membranes are incubated with a solution of $mIgGQD_{630}$ in PBS (1), 50 % human serum (2) and 50 % IgG depleted human serum (3) d) Immuno dot blot analysis of IgG (Fc) recognition by immuno-QDs ($mIgGQD_{630}$) incubated in 10 % or 50 % IgG depleted HS. $mIgGQD_{630}$ have been spotted on the PVC membrane. Afterwards the membranes were incubated with the 100 nm fluorescent SiO_2@HS NPs in PBS, 10 % and 50 % IgG depleted HS. The IgG epitope recognition is decreasing proportionally to the amount of proteins in the media.

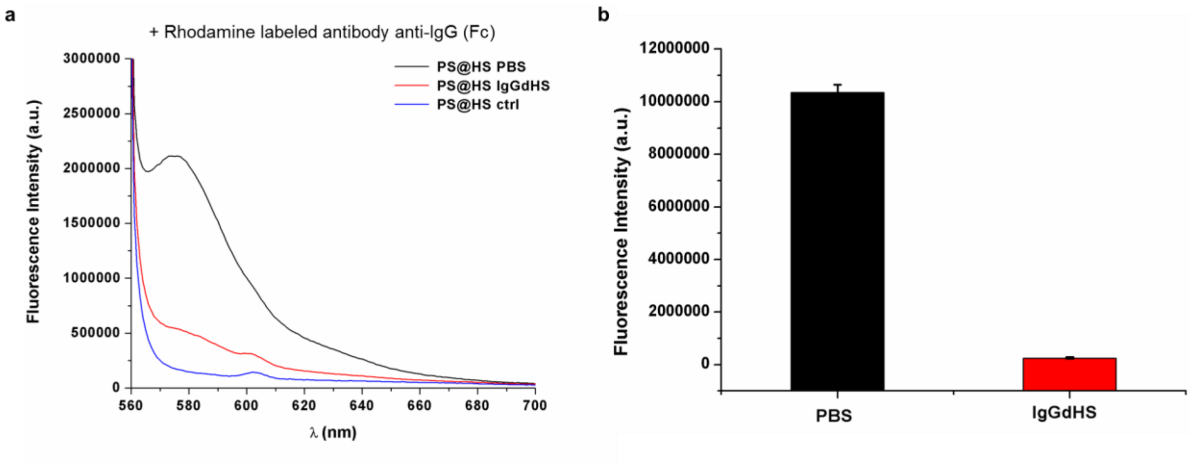

Supplementary Figure 23. Exposure of IgG epitopes on PS NPs in PBS and *in situ* (in IgG depleted serum) using a different immunoprobe, consisting of Rhodamine conjugated IgG antibody. a) Fluorescence spectra of the 200 nm PS@HS NPs labeled with Rhodamine anti IgG (Fc) antibody in PBS (black line) and under 50% depleted IgG human serum (red line). It shows a strong reduction of the interactions in complex biological media.

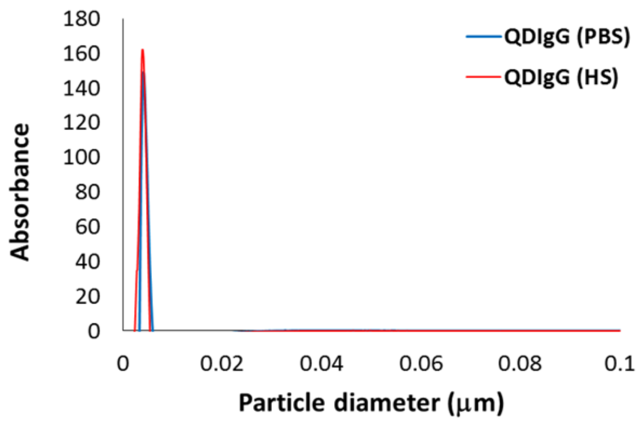

Supplementary Figure 24. Characterization of QDs by differential centrifugal sedimentation (DCS). Size distribution comparison QDsIgG dispersed in PBS (blue line) and QDsIgG dispersed in 50% depleted IgG human serum (red line).

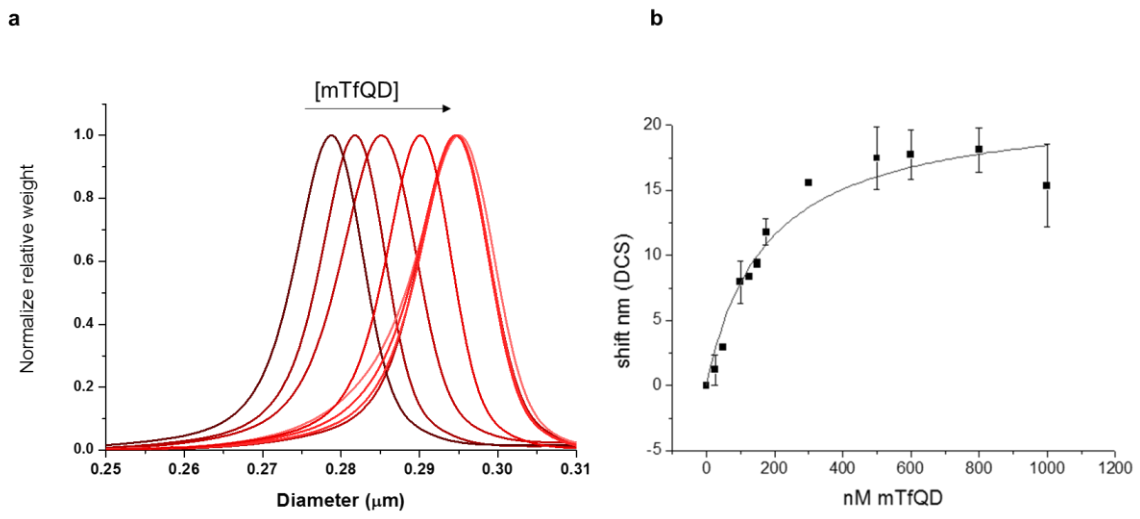

Supplementary Figure 25. Biorecognition assay by differential centrifugal sedimentation. a) DCS graph of PS@Tf NPs (200 nm diameter) labeled with mTfQD. b) DCS data fitting analysis of PS@Tf NPs (200 nm diameter) labeled with $mTfQD_{630}$.

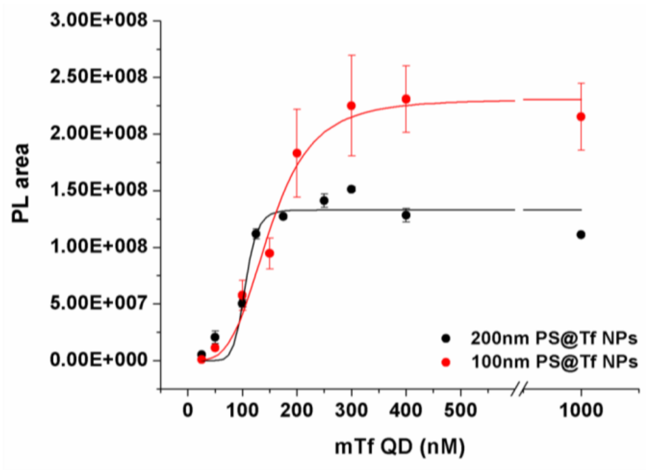

Supplementary Figure 26. Fluorescence spectroscopy data fitting analysis of PS@Tf NPs (0.5 $mg·mL^{-1}$ for 200 nm NPs and 1 $mg·mL^{-1}$ for 100 nm NPs) labeled with $mTfQD_{630}$.

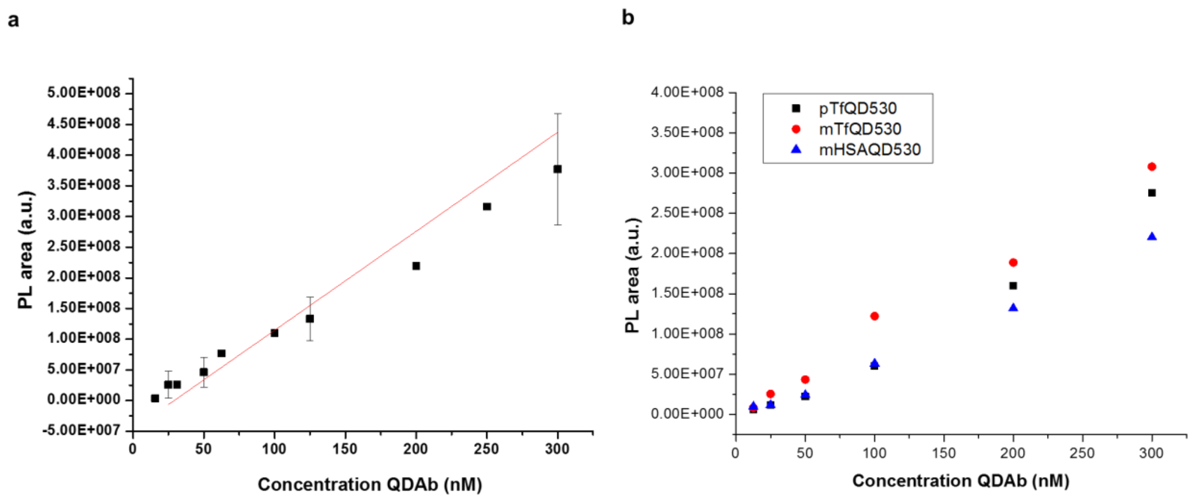

Supplementary Figure 27. Calibration curve of QDAb by steady-state fluorescence spectroscopy. a) Calibration curve for $mTfQD_{630}$ (different batches of $mTfQD_{630}$ from the same batch of QD_{630}). Linear fitting analysis ($y = ax + b$, a = 1.61e06, b = -4.67e07 and R-square 0.98). b) Calibration curve for QD_{530} (functionalized with pTf, mTf and mHSA). Linear fitting analysis for: $pTfQD_{530}$ $y = ax + b$, a = 9.75 e05, b = -2.53 e07 and R-square 0.99, $mTfQD_{530}$ $y = ax + b$, a = 1.05 e06, b = -2.59 e06 and R-square 0.99, and $mHSAQD_{530}$ $y = ax + b$, a = 7.57 e05, b = -1.2 e07 and R-square 0.99.

	DCS		NTA	
	Size (weight)	Size (number)	Size	NPs $\cdot mL^{-1}$
**200 nm PS**	205 nm	204 nm	239 nm	$(2.9 \pm 0.9) \cdot 10^{11}$
**200 nm PS@Tf**	283 nm	282 nm	271 nm	$(1.7 \pm 0.9) \cdot 10^{11}$
**100 nm PS**	100 nm	97 nm	93 nm	$(2.8 \pm 1) \cdot 10^{12}$
**100 nm PS@Tf**	141 nm	141 nm	111 nm	$(8.2 \pm 1.3) \cdot 10^{11}$
**200nm PS**	205 nm	204 nm	171 nm	$(2.8 \pm 0.2) \cdot 10^{11}$
**200 nm PS@HS**	230 nm	230 nm	221 nm	$(4.4 \pm 1) \cdot 10^{11}$
**100 nm SiO_2**	95 nm	79 nm	109 nm	$(5.5 \pm 0.5) \cdot 10^{11}$
**100 nm SiO_2@HS**	78 nm	76 nm	121 nm	$(4.6 \pm 0.7) \cdot 10^{11}$

Supplementary Table 1. Size distribution and concentration of bare and Tf coated PS NP (100 and 200 nm), and bare and 80 % HS corona coated PS NPs (200 nm) and SiO_2 NPs (100 nm). Both DCS and the NTA data shows an increase in size of both the PS NPs and the SiO_2 NPs upon adsorption of the biomolecular corona. NTA has also been used to measure the concentration before and after the preparation of the biomolecular corona.

DLS characterization				
	Z-Average	PDI	Intensity	Number
**200 nm PS**	240 nm	0.02	250 nm	219 nm
**200 nm PS@Tf**	259 nm	0.015	269 nm	237 nm
**100 nm PS**	107 nm	0.02	112 nm	87 nm
**100 nm PS@Tf**	122 nm	0.06	130 nm	97 nm
**200nm PS**	181 nm	0.03	191 nm	157 nm
**200 nm PS@HS**	203 nm	0.02	211nm	184 nm
**100 nm SiO_2**	112 nm	0.02	117 nm	92 nm
**100 nm SiO_2@HS**	130 nm	0.05	138 nm	105 nm

Supplementary Table 2. Dynamic light scattering measurements of the bare and Tf coated PS NP (100 nm and 200 nm), and bare and 80 % HS corona coated PS NPs (200 nm) and SiO_2 NPs (100 nm). The DLS data confirm the DCS and NTA analysis. The position of the peak for all NPs shifts toward higher hydrodynamic diameters upon adsorption of the human serum biomolecules.

PS NP			SiO$_2$ NP		
**MW**	**Protein identity**	**NSpC**	**MW**	**Protein identity**	**NSpC**
69367	Serum albumin	9.24	30778	Apolipoprotein A	11.14
36154	Apolipoprotein E	9.22	36154	Apolipoprotein E	9.48
41737	Actin cytoplasmic	7.98	9332	Apolipoprotein C	7.06
54306	Vitronectin	7.55	13227	Ig heavy chain V-III region	5.98
37651	Complement factor H-related protein	7.49	69367	Serum albumin	5.89
103357	Inter-alpha-trypsin inhibitor	4.71	37651	Complement factor H-related protein	4.90
45675	Plasma serine protease inhibitor	4.49	14747	Serum amyloid A-4 protein	4.47
11609	Ig kappa chain C region	4.42	70109	Coagulation factor XI	4.33
70109	Coagulation factor XI	4.39	21276	Apolipoprotein D	3.72
59578	Histidine-rich glycoprotein	4.30	54306	Vitronectin	3.64
13227	Ig heavy chain V-III	3.88	37655	Ig alpha-1 chain C region	3.50
49307	Ig mu chain C region	3.64	67792	Coagulation factor XII	3.50
43174	Selenoprotein P	3.56	11609	Ig kappa chain C region	3.41
187147	Complement C3	2.74	16228	Ig heavy chain V-II region	3.25
37655	Ig alpha-1 chain C region	2.72	46737	Alpha-1-antitrypsin	2.82
38298	Beta-2-glycoprotein	2.68	43174	Selenoprotein P	2.75
67792	Coagulation factor XII	2.65	45205	Haptoglobin	2.63
55154	Plasma protease C1 inhibitor	2.32	77064	Serotransferrin	2.57
85697	Gelsolin	2.09	187147	Complement C3	2.47
25039	C-reactive protein	2.05	59578	Histidine-rich glycoprotein	2.43
39325	Alpha-2-HS-glycoprotein	1.96	49307	Ig mu chain C region	2.41
14707	Ig kappa chain V-II region	1.74	57833	Clusterin	2.28
47651	Alpha-1-antichymotrypsin	1.08	192784	Complement C4-A	1.30
226530	Myosin-9	1.02	85697	Gelsolin	1.23
123799	Vinculin	0.83	151076	Proteoglycan	1.22
192750	Complement C4	0.66	515611	Apolipoprotein B-100	0.90
85533	Complement factor B	0.60	163290	Alpha-2-macroglobulin	0.73

Supplementary Table 3. Major Hard Corona Proteins of 200 nm PS NP and 100 nm SiO$_2$ NPs incubated with 80 % of HS identified by LC-MS. NSpC: Normalized spectral count (NSpC) values were calculated for each protein hit according to equation 1.

Supplementary references:

1. Salvati, A. et al. Transferrin-functionalized nanoparticles lose their targeting capabilities when a biomolecule corona adsorbs on the surface. *Nature Nanotech.* **8**, 137-143 (2013).
